# Comparison of two different ecological floating bio-reactors for pollution control in hyper-eutrophic freshwater

**DOI:** 10.1038/s41598-018-32151-5

**Published:** 2018-09-24

**Authors:** Naxin Cui, Guifa Chen, Yaqin Liu, Li Zhou, Min Cai, Xiangfu Song, Guoyan Zou

**Affiliations:** 1 Eco-environmental Protection Research Institute, Shanghai Academy of Agricultural Sciences, Shanghai, 201403 PR China; 2 Shanghai Engineering Research Centre of Low-carbon Agriculture (SERCLA), Shanghai, 201415 PR China

## Abstract

The use of ecological floating beds (EFBs) to control water pollution has been increasingly reported worldwide due to the severe situation of eutrophication in water bodies. In this study, two kinds of EFBs were set up under similar condition to compare their purification efficiency in hyper-eutrophic water. The conventional ecological floating bed (CEFB) was made of polystyrene foam board, and the enhanced ecological floating bio-reactor (EEFB) was designed as an innovative hollow, thin floating bed integrated with substrates of zeolite and limestone. The results showed that the EEFB increased treatment efficiency of total nitrogen (TN), total phosphate (TP), and ammonia nitrogen (NH_4_^+^-N) to 63.5%, 59.3%, and 68.0%, respectively. Plant accumulation was the main pathway for TN and TP removal in the CEFB. Microbial degradation played an increasingly important role in TN and TP removal in the EEFB. A higher concentration of nitrogen cycling bacteria was recorded in the EEFB than the CEFB (*P* < 0.05), suggesting that the substrates might enhanced the removal efficiency of the EEFB by promoting the growth of microorganisms rather than their absorption effect.

## Introduction

Hyper-eutrophication leads to harmful cyanobacterial blooms, which is increasing worldwide, and represents a serious threat to drinking water supplies and the ecological and economical sustainability of freshwater ecosystems^1,2^. In China, lakes became eutrophic (66%) or hyper-eutrophic (22%) due to increasing discharge of industrial, agricultural and/or domestic wastewater^3^. Nitrogen (N) and phosphorous (P) are identified as the two major nutrients which should be controlled for decreasing the occurrence of harmful blooms and mitigating the serious situation of eutrophication^2,4^. In controlling eutrophication, the researches focused on ecological technologies such as artificial constructed wetlands and ecological floating beds (EFBs) for water purification in rivers or lakes^5,6^. However, problems of clogging in the substratum layer^7^ and the larger cover area usually restrict the usage of constructed wetlands^8,9^. EFBs with the advantage of low cost, effectiveness and better plant accommodation, have been widely used as an *in-situ* ecological remediation technology for treating surface water in Japan^10^, Australia^11^, England^12^, the United States^13^, Italy^14^, China^9,15,16^ and so on.

For conventional EFBs (CEFBs), the assimilation of N and P by plants growth plays important role in nutrient removal of eutrophic water bodies. Its performance was affected by the growth rate and limited stand biomass of the plants in the beds^15^. The addition of biofilm carriers such as a semi-soft assembly medium, plastic filling, and rice straw to planted floating beds could enhance the pollutant removal efficacy^17,18^. The biofilm carriers which are hung below the EFBs provide additional surfaces for the attachment of microorganisms which contribute to pollutant removal. As a common substrates that have been widely used in constructed wetlands, zeolite and limestone possess a good adsorption capacity for nitrogen and phosphate, and also provide micro-environmental conditions for the growth of microorganisms. In the present paper, an enhanced EFB (EEFB) was designed by innovatively introducing the substrate mixture of zeolite and limestone to improve its purification efficacy when utilized in hyper-eutrophic water. Experiments were conducted to explore the nutrient removal mechanism in the EFBs. The objectives of this study are: (1) investigating the nutrient removal performance of the EEFBs that employ substrates in comparison with the CEFBs; (2) measuring the plant growth and the major microorganism related to nitrogen and phosphorus removal; and (3) exploring the pathway of nutrient removal in the EFBs system.

## Results

### Nutrient removal efficacy

**Figure 1:**
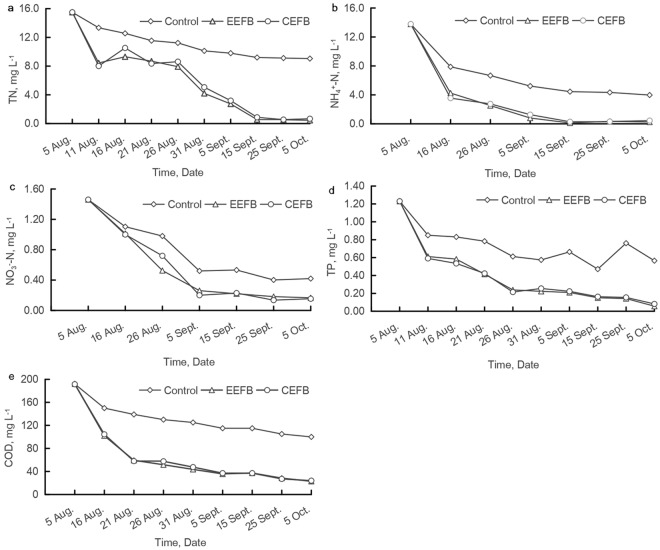
Time courses of different nutrient concentrations in the water column in the EEFB, the CEFB and the control. (**a**) TN; (**b**) NH_4_^+^-N; (**c**) NO_3_^−^-N; (**d**) TP; (**e**) COD.

At the end of the experiment, the concentrations of total nitrogen (TN), ammonia nitrogen (NH_4_^+^-N), total phosphate (TP), and chemical oxygen consumption (COD) in the EEFB were lower than those in the CEFB (Fig. 1a,b,d,e). However, the concentration of nitrate nitrogen (NO_3_^-^N) in the EEFB was slightly higher than that in the CEFB (Fig. 1c). The TN concentrations in the EEFB and CEFB decreased from 15.50 mg L^−1^ to 0.45 mg L^−1^ and 0.67 mg L^−1^, respectively (Fig. 1a). The initial concentration of TP was 1.23 mg L^−1^. At the end of the experiment, the final concentration of TP in the EEFB reached the lowest at 0.077 mg L^−1^ in water column, followed by the CEFB and the control at 0.57 mg L^−1^, approximately 7 times higher (Fig. 1d). For NH_4_^+^-N and COD, the EEFB showed the dramatic decrease from the initial concentrations of 13.78 and 191.2 mg L^−1^ to the final concentrations of 0.06 and 22.90 mg L^−1^ in the water column, respectively (Fig. 1b,e).

The TN removal rate in the EEFB and CEFB were 82.8% and 70.7%, respectively (Table 1). The TN removal rate in the control was only 19.7%. The removal rate of TN in the EEFB was significantly higher than that of the CEFB (*P* < 0.05). The net removal rate of TN in the EEFB and CEFB were 63.1% and 51.0%. The removal rate of NH_4_^+^-N in the EEFB and CEFB were 91.5% and 81.2%, significantly higher than that of the control (*P* < 0.05) (Table 1). No significant difference was found between the net removal rate of NH_4_^+^-N in the EEFB and CEFB (*P* = 0.221).

**Table 1:** The removal efficiency of TN, TP and NH_4_^+^-N in different treatments. Data are means followed by standard errors (±SE) (n = 3).

The treatment	TN		TP		NH_4_^+^-N	
	Removal rate (%)	Net removal rate (%)	Removal rate (%)	Net removal rate (%)	Removal rate (%)	Net removal rate (%)
The control	19.4 ± 1.68a	—	14.6 ± 1.13a	—	23.5 ± 1.71a	
EEFB	82.8 ± 2.48b	63.4 ± 1.21a	73.9 ± 2.08b	59.3 ± 1.37a	91.5 ± 4.25b	68.0 ± 2.37a
CEFB	70.7 ± 1.76c	51.3 ± 1.02b	67.5 ± 1.87b	52.8 ± 2.18a	83.2 ± 3.64b	57.7 ± 2.02a

Different letters indicate significantly different among three treatments at *P* ≤ 0.05.

TP was removed at 73.9% in the EEFB, higher than 67.5% removal rate of TP in the CEFB (Table 1). The TP removal rate in the control was only 14.6%. The TP net removal rate in the EEFB and the CEFB was 59.3% and 52.8%, respectively.

### The biological characteristics and nutrient content in different plant tissues of *Cyperus alternifolius* L

The plant biological characteristics are reported in Table 2. The plants in the two treatment systems all thrived. The total dry biomass of the plants in the EEFB was 1.13 ± 0.10 kg m^−2^, which was about 6.28 times higher than the initial plant biomass. A higher total dry biomass was recorded in the CEFB, weighting at 1.32 ± 0.23 kg m^−2^ and was about 7.33 times higher than the initial plant biomass. However, no significant difference was recorded in both the total dry biomass (*P* = 0.254) and the relative growth rate (*P* = 0.315) between the EEFB and CEFB. The aboveground dry biomass comprised the most weight of the total dry biomass, which accounted for 78.0% in the EEFB and 81.8% in the CEFB, respectively. The plant height was significantly higher in the CEFB than those in EEFB (*p* < 0.05); however, the root length and underground dry biomass of the plants in the EEFB were considerably higher than those in the CEFB (*P* < 0.05).

**Table 2:** The growth characteristics of *C. alternifolius* in EEFB and CEFB. Data are means followed by standard errors (±SE) (n = 3).

The treatment	Dry biomass kg m^−2^					
	Total	Aboveground	Underground	Plant height (cm)	Root length (cm)	RGR
The initial	0.18 ± 0.03a	0.13 ± 0.03a	0.05 ± 0.02a	72.90 ± 9.63a	24.00 ± 4.60a	
EEFB	1.13 ± 0.10b	0.87 ± 0.07b	0.26 ± 0.08b	99.50 ± 12.03b	57.75 ± 6.08b	0.0329 ± 0.0035a
CEFB	1.32 ± 0.23b	1.08 ± 0.21b	0.25 ± 0.05b	125.25 ± 5.69c	43.67 ± 9.44c	0.0366 ± 0.0078a

RGR: relative growth rate. Different letters indicate significantly different among three treatments at *P* ≤ 0.05.

The nutrient content of the different plant tissues varied at the beginning and the end of the experiment (Table 3). The initial N and P content in the buds were highest at 49.42 ± 15.59 mg g^−1^ N and 3.20 ± 0.42 mg g^−1^. However, the N content in the leaves was highest at 37.95 ± 12.14 mg g^−1^ at the end of the experiment in the CEFB and 37.12 ± 14.66 mg g^−1^ in the EEFB. The buds had the highest P content of 2.92 ± 0.72 mg g^−1^ in the EEFB and 2.76 ± 0.22 mg g^−1^ in the CEFB.

**Table 3:** Changes of nutrient contents in different plant organs of *C. alternifolius* in EEFB and CEFB. Data are means followed by standard errors (±SE) (n = 3).

	N content (mg g^−1^)				P content (mg g^−1^)			
	Leave	Stem	Bud	Root	Leave	Stem	Bud	Root
Initial content	48.86 ± 10.73	30.42 ± 8.90	49.42 ± 15.59	39.75 ± 9.82	2.17 ± 0.27	2.49 ± 0.73	3.20 ± 0.42	2.68 ± 0.74
EEFB	37.12 ± 14.66	18.11 ± 4.70	28.01 ± 7.45	20.66 ± 7.72	1.75 ± 0.35	1.76 ± 0.56	2.92 ± 0.72	1.60 ± 0.36
CEFB	37.95 ± 12.14	25.90 ± 9.43	36.39 ± 12.56	24.91 ± 9.83	1.99 ± 0.06	1.80 ± 0.05	2.76 ± 0.22	1.74 ± 0.45

**Figure 2:**
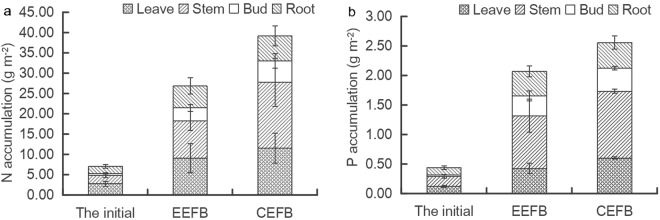
Accumulation of N and P in different organs of *C. alternifolius*. Data are means followed by standard deviation (±SD) (n = 3). (**a**) N accumulation; (**b**) P accumulation.

At the end of the experiment, the stems accumulated for the highest amount of N with 9.18 and 16.29 g m^−2^, accounting for 34.15% in the EEFB and 41.52% in the CEFB, followed by the leaves, roots, and buds (Fig. 2a). The aboveground tissues accumulated 21.52 and 33.04 g m^−2^ of N, accounting for 80.06% and 84.24% in the EEFB and the CEFB, respectively (Fig. 2a). Less than 20% of N accumulated in the underground plant tissue. P shared a similar accumulation pattern in the different plant tissues in both the EEFB and the CEFB (Fig. 2b). The stems accumulated 0.89 and 1.13 g m^−2^ of P, followed by the leaves, the roots and the buds. The aboveground biomass accumulated 1.66 and 2.13 g m^−2^ of P, accounting for around 80% of P and only 20.08% and 16.83% of P accumulated in the underground plant tissues in the EEFB and the CEFB, respectively (Fig. 2b).

### Microorganism composition changes for nitrogen cycling bacteria

**Figure 3:**
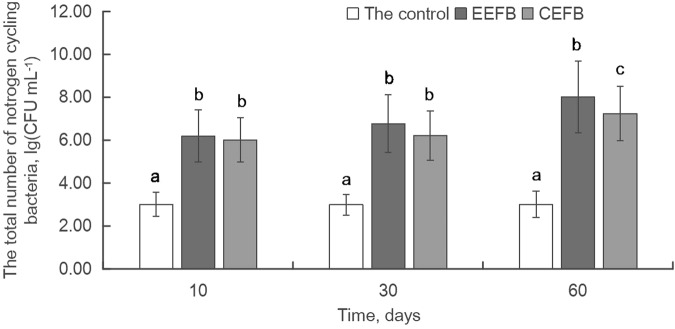
Time courses of total number of nitrogen cycling bacteria in the water column of the control, EEFB and CEFB. Data are means followed by standard deviation (±SD) (n = 3). Different letters indicate significantly different among three treatments at *P* ≤ 0.05.

The total number of nitrogen cycling bacteria in the EEFB and CEFB was significantly higher than that in the control in 10 days after the experiment (*P* < 0.01). It increased in the EEFB and CEFB continually in the experiment, and was highest in the EEFB, followed by the CEFB and the control at different testing times (Fig. 3). At the end of the experiment, the total number of nitrogen cycling bacteria in the EEFB was significantly higher than that in the CEFB (*P* < 0.05). The total number of nitrogen bacteria in the EEFB and CEFB had grown three orders of magnitude than that of the control at 10 days after the experiment began. The highest increase in the total number of nitrogen bacteria was recorded in the EEFB (6.21 to 8.03), followed by the CEFB (6.03 to 7.26) during the experiment. However, the total number of nitrogen bacteria decreased somewhat at 30 days, and became almost equal to that of 10 days at the end of the experiment in the control(Fig. 3).

**Figure 4:**
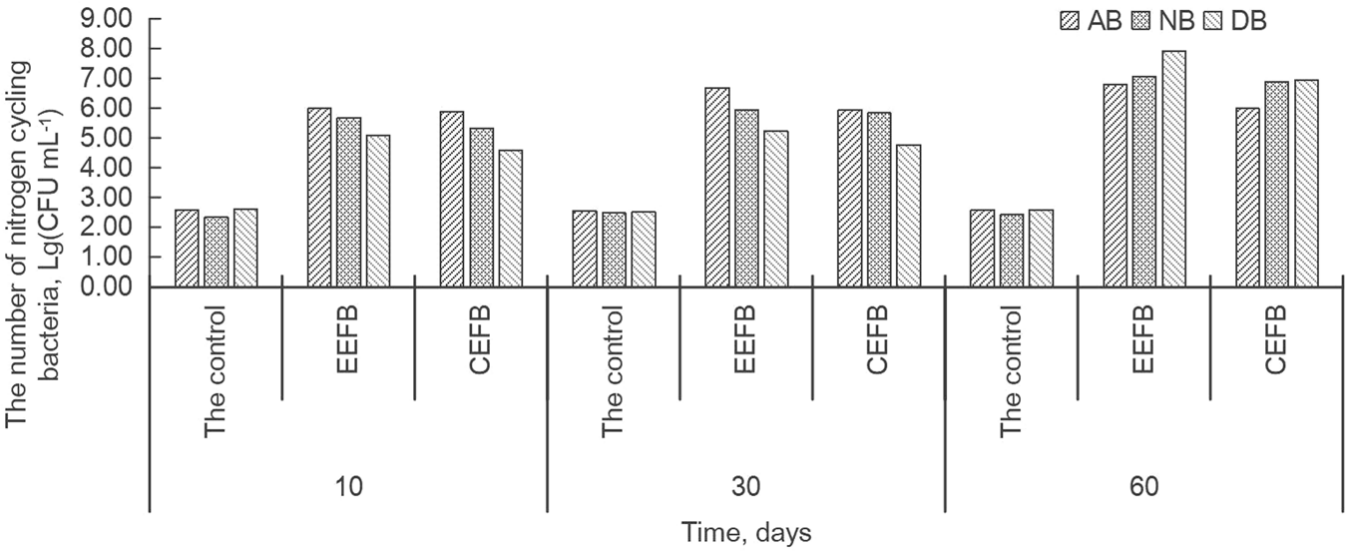
Time courses of different microorganisms for nitrogen cycling in the control, EEFB and CEFB. AB: ammoniating bacteria, NB: nitrifying bacteria, DB: denitrifying bacteria.

The number of different kinds of nitrogen cycling bacteria increased as the experiment continued, especially the number of denitrifying bacteria (DB), which increased by more than two orders of magnitude, from 5.11 to 7.94 in the EEFB, and from 4.60 to 6.95 in the CEFB (Fig. 4). In the EEFB, the number of ammonifying bacteria (AB) was highest with a 6.00 value at 10 days and 6.70 at 30 days, followed by the nitrifying bacteria (NB), while the number of DB was the lowest. However, the number of DB became the highest at the end of the experiment, followed by the NB and the AB (Fig. 4). In the CEFB, the concentrations of the different kinds of nitrogen cycling bacteria shared a similar changing curve along the time of experiment with those in the EEFB. It revealed that the AB was the dominant nitrogen cycling bacteria at the beginning and mid-term of the experiment and the DB became the dominant one at the end of the experiment. Little change was found in the number of different nitrogen bacteria during different testing time in the control.

### The nitrogen and phosphorus removal pathway

The respective quantities of N removed in EEFB and CEFB were 17.33 g and 16.27 g, around 4 times higher than that in the control. In the EEFB, microorganisms degraded most of the removed N, accounting for 49.86%. The contribution of N removal by plant uptake was 46.27%, and only 3.88% of N was removed by substrate absorption (Table 4). However, in the CEFB, plant uptake contributed the highest proportion of N removal, accounting for 80.53%. The microorganism contribution in N removal was less than 20%. Only 4.05 g of N and 0.24 g of P were removed in the control, which was mainly attributed to microbial degradation.

**Table 4:** The pathway of N and P removal in different treatments.

Treatment	Pathway of N removal (%)			Pathway of P removal (%)		
	Plant removal	Substrate removal	Microbial degradation	Plant removal	Substrate removal	Microbial degradation
The control	—	—	100.00	—	—	100.00
EEFB	46.27	3.88	49.86	53.72	2.28	44.00
CEFB	80.53	—	19.47	76.75	—	23.25

Total 1.23 g of P was removed in the EEFB during the experimental period. Plant uptake removed most of the P (0.66 g, accounting for 53.72%), followed by microbial degradation (0.54 g, accounting for 44.00%). The substrates removed only 2.28% of the total P removal in the EEFB (Table 4). The amount of P removed by the CEFB was 1.12 g, in which the plant uptake removed 0.86 g of P, accounting for 76.25%. Only less than one-quarter of P was removed by microbial degradation in the CEFB.

## Discussion

It was reported that EFBs are potential alternatives to traditional constructed wetlands for remediation of nutrient-rich water^8^. The floating beds vegetated with a terrestrial Italian ryegrass could remove almost completely all the NO_3_^−^-N with an initial concentration up to 150 mg L^−1^ in 10 days^14^. In our experiment, the CEFB planted with an aquatic *C. alternifolius* removed almost 89.7% of the NO_3_^−^-N from the water column with a low initial concentration of 1.46 mg L^−1^. A similar removal rate of 89.9% for NH_4_^+^-N was also observed (Table 1). It showed that the planted floating beds could effectively remove the nitrogen nutrients in the water column. More than 90% of the TP in the water column was removed with an initial concentration of 1.23 mg L^−1^ in the EFBs in our study, which was higher than the removal efficacy of 45–75% reported by White and Cousins^19^. This difference may be attributed to various initial concentration (0.08–0.22 mg L^−1^) and different plants vegetated (*Canna flaccida* and *Juncus effusus*)^19^. An additional sorbent/substrate surrounding the root system of the plants in floating treatment wetlands was needed to adequately remove nutrients^20,21^. The EEFBs integrated with different substrates have been reported to improve purification efficiency^22,23^. In our study, although both CEFB and EEFB effectively removed N, P and COD in the water, the EEFB purification efficiency was significantly enhanced (*P* < 0.05), especially for the removal of TN and NH_4_^+^-N (Fig. 1). The results showed that adding substrates such as zeolite and limestone to EFBs could effectively improve the nutrient removal efficiency.

The addition of various substrates such as rice straw, plastic filling and ceramsite to the EFBs could enhance purification efficacy, since the substrates would benefit the growth of the macrophytes, and improve the biomass and the activity of the microorganisms^22,24,25^. In our present study, however, *C. alternifolius* was found to grow better in the CEFB without the addition of substrates. It had a higher dry biomass (both the aboveground and the total biomass), plant height, and RGR in the CEFB than in the EEFB (Table 2). Plant uptake played a major role in the removal of N and P from the water^26,27^. In the CEFB, the main pathway of nutrient removal was plant uptake with 80.53% of TN removal and 76.75% of TP removal (Table 4). The increase in plant biomass contributed to nutrient accumulation in the plant, which explained higher growth rate of *C. alternifolius* and higher N and P removal quantity by plant uptake in the CEFB than that in the EEFB (Fig. 2). Although the contribution of plant uptake to nutrient removal in the EEFB was lower than that in the CEFB, more N and P were removed in the EEFB than in the CEFB (Table 1). The plants in the EFBs could not only directly transfer the nutrients into its biomass from the water column, their root systems could also create a variety of microenvironments for microorganisms to breed and reproduce. This favored the degradation of organic matters and other nutrients^17,28^. *C. alternifolius* in the EEFB developed longer roots to absorb sufficient nutrients from the water column with low-nutrient for growth^19^. Longer roots may have favored the growth of microbes in the root zone^29^. The addition of substrates in the EEFB systems could also stimulate the growth of microorganisms. The contribution of substrates to the total nutrient removal was less than 4% (Tables 3 and 4), indicating that the substrate absorption was not the main cause of the higher purification efficacy in the EEFB. Higher total amount of nitrogen cycling bacteria and different kinds of nitrogen bacteria were recorded in the EEFB than in the CEFB (Figs 3 and 4). During the early days of the experiment, higher concentration of AB was enhanced by the existence of soluble organic nitrogen and higher concentration of NH_4_^+^-N in the water column promoted the growth of NB in both the EEFB and the CEFB (Figs 1b and 4). The ammonification, nitrification might be the main cause of nitrogen removal. NH_4_^+^-N in the water can be converted into NO_2_^−^-N and NO_3_^−^-N under aerobic conditions by NB, and then the NO_2_^−^-N and NO_3_^−^-N are transformed into N_2_ or N_2_O by DB under the condition of anoxia and escape from the water^23,29^. Along with the experiment continuing, NH_4_^+^-N was transformed by NB to NO_3_^−^-N, which contributed to the growth of DB and the cause of denitrification^29^. Higher number of nitrogen cycling bacteria enhanced its nitrogen removal rate comparing to the CEFB. Microbial degradation accounted for almost half of the total N removal (Table 4). This showed that microbial nitrification and denitrification were the main nitrogen removal routes in the EEFB^4,22,30^. The effects of the plants and substrates on the higher purification efficacy of N were primarily indirectly obtained by favoring the growth of microorganisms through providing various micro-environments in the EEFB. Plant uptake was the main removal pathway of P in the EEFB and the CEFB, which is consistent with the result of Zhang *et al*.^23^. In our study, around 80% of N and P absorbed by the plant accumulated in the aboveground biomass, which indicates that harvesting the aboveground tissues of the plants is an effective method to remove most of the nutrients in polluted water^23,26^. However, White and Cousins^19^ reported that nearly half of the nutrients accumulated within the whole plant were accumulated within the plant root systems and suggested whole-plant harvest. Therefore, the plant harvest management should be considered in the further research of nutrient accumulation in different plant tissues.

## Conclusions

In this study, a kind of EEFB was designed. Its efficacy of water purification was analyzed comparing to the CEFB when applied to water pollution control in hyper-eutrophic water. The EEFB treatment system had a higher purification efficacy than the CEFB for pollution control in hyper-eutrophic water. The substrates in the bioreactor could provide a habitat for microorganisms and improve the removal efficiency of nutrients in the water. Microbial degradation was the main pathway of N reduction in the EEFB. *C. alternifolius* plant uptake played a major role in P removal in the EEFB and the CEFB.

## Materials and Methods

### Ecological floating bed design and experiment set-up

**Figure 5:**
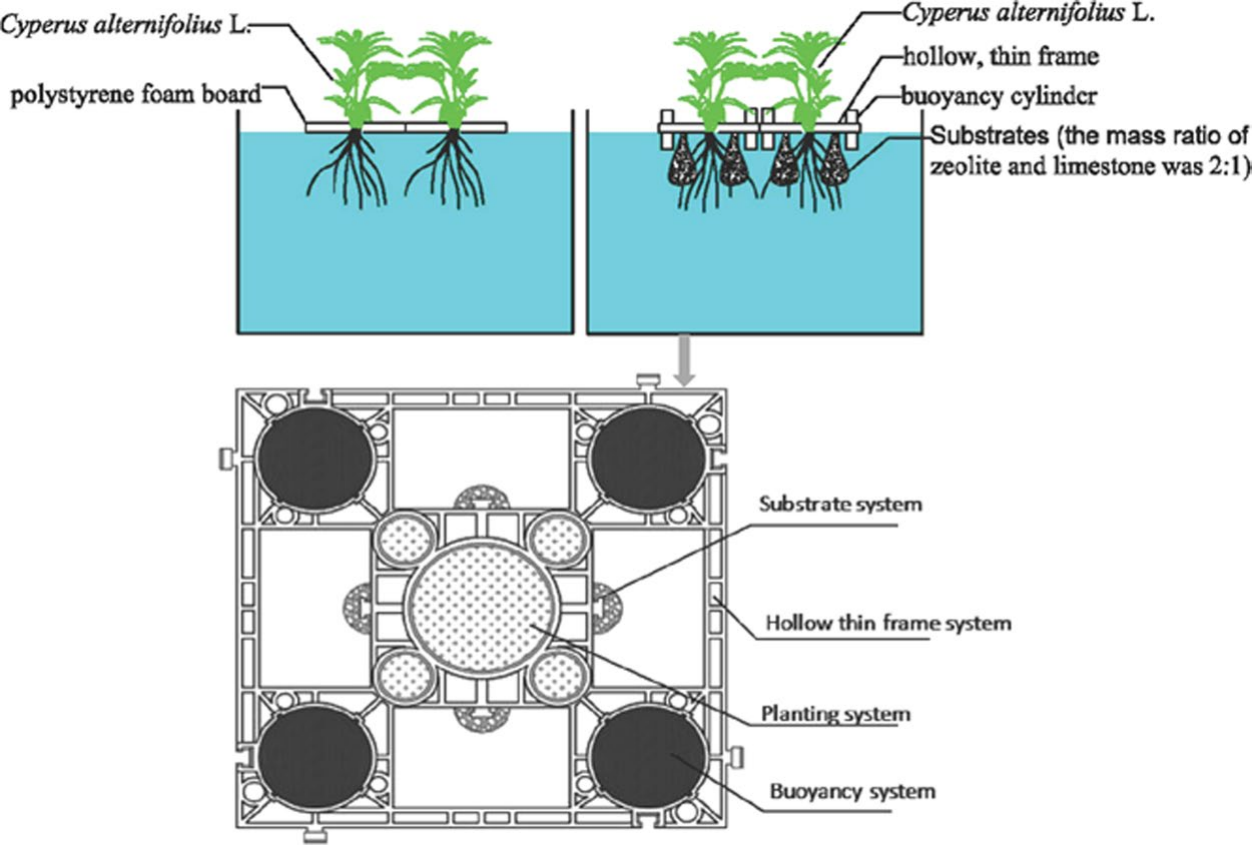
Schematic diagram of the ecological floating beds (EFBs).

In this research, two types of EFBs with the same surface area of 0.2025 m^2^ were designed to compare their purification efficiency in hyper-eutrophic waters under similar conditions. The CEFB was made of polystyrene foam board in a length of 45 cm, a width of 45 cm and a thickness of 4 cm. There was a planting hole in a diameter of 13 cm at the center of the board. The EEFB, primarily made of PVC (Fig. 5), was an innovatively hollow, thin skeleton with a dimension in length of 45 cm, a width of 45 cm and a thickness of 3 cm (Fig. 5). There was a planting hole in a diameter of 13 cm at the center of the EEFB skeleton. Four nylon bags of substrates were hanged to the four sides of the inner square frame (25 cm in width) of the EEFB, respectively (Fig. 1). Each bag of the substrates weighed two kilograms. Before placed in the nylon bag, the zeolite and limestone (the mass ratio was 2:1) substrates in the diameter of 2–4 mm were washed with deionized water. Four hollow plastic buoyancy cylinders in a diameter of 10 cm and a height of 25 cm were arranged at the four corners of the EEFB. The plant *C. alternifolius* was used in the experiment. The seedlings were cultivated at 10 days prior to the experiment. Seedlings at similar height of 72.9 cm and fresh weight of 530.6 g were chosen. One seedling was planted in the hole of each floating bed.

The mesocosm experiment was performed in nine rectangular concrete ponds with identical inner dimensions of 1.5 m in length, 1.0 m in width and 1.0 m in depth. The concrete ponds were filled with 1350 L of hyper-eutrophic water which were prepared by adding ammonium nitrate, potassium dihydrogen phosphate, and glucose into 1/40 concentration of the Hoagland nutrient solution. The concentrations of TN, TP, NH_4_^+^-N and COD in the experimental water were 15.50 mg L^−1^, 1.23 mg L^−1^, 13.78 mg L^−1^, and 192 mg L^−1^, respectively. There were three treatments in this experiment with three replicates. The treatments with two units of planted floating foam bed and two units of enhanced floating bed are in the CEFB and EEFB, respectively. The pond with only the experimental water served as the control. The coverage rate of the EFBs in the ponds was 26.0%. All the concrete ponds were under a plastic roof shed to facilitate natural photoperiods and to avoid the effects of rainfall. The experiment was conducted at the Zhuanghang Integrated Experiment Station (E121°23′, N30°53′), located in Shanghai city of the Yangtze River delta, China from August 5, 2013 to October 5, 2013. During the period of the experiment, the water loss from evaporation and transpiration was supplied by adding deionized water to the original level every two or three days.

### Sampling and Analysis of water, plants, and substrate

Water was sampled from each pond every five days in the first month and every ten days in the second month. A total of five sub-samples (200 mL) were collected from five different spots at four corners and the center at the middle water depth of the pond. Five sub-samples of water from different sampling spots were mixed together to form one water sample. The water samples were analyzed for TN, NH_4_^+^-N, NO_3_^−^-N, and TP using a flow-injection auto-analyzer (Seal, AA3 Analyzer, Germany). COD was analyzed using the National Environmental Protection Agency of China standard protocol (2002). Partial water samples at 10 days, 30 days, and 60 days were used for the microorganism analysis. The most probable number (MPN) method was used to determine the number of AB, NB, and DB in the water samples^31^. The culture medium for AB, NB and DB were prepared using the method of Ma *et al*.^31^. All MPN tubes were incubated at the temperature of 30 °C and checked for nitrogen cycling bacteria growth after 14 d. MPN values were calculated from standard MPN tubes^32^, and recorded as CFU/mL. The number of the bacteria was transformed by log_10_(CFU/mL). All of the sediment was collected and air dried at room temperature, and the N and P contents in the sediment was tested with the method described by Lu^33^.

To quantify the plant growth in the EFBs, three plants at similar height and weight compared to the seedlings planted in the floating beds were randomly selected and tested when the experiment was initiated. After washing with deionized water and drying with absorbent paper, the plants were weighed on an electronic scale to determine their fresh weight (FW). The roots, stems, and leaves were separated to record the fresh weight and then dried to a constant weight after 48 hours of oven-drying at 80 °C to determine the dry weight (DW). The TN and TP content of the dried plant tissues were measured with the H_2_SO_4_-H_2_O_2_-Colorimetric method^34^. At the end of the experiment, the plants in the EFBs were collected, and the same parameters were measured using the same methods.

TN and TP content of the substrates were measured at both the beginning and end of the experiment. The substrates were dried at room temperature and passed through a 100-mesh screen. Five grams of the substrate samples were soaked in 100-mL deionized water and rotated in the shaker at a speed of 160 rpm for 48 h to extract nutrients absorbed in the substrate. The flask mixture was then filtered to measure the TN and TP concentrations of the filtrate.

### Data Analysis

All of the data analyses were performed in triplicate, and the data were expressed as mean ± standard errors. The removal rates (R, %) within each treatment and the relative growth rate (RGR) of the plants were analyzed according to the following formulas.

1 $${\rm{R}}=100\times ({{\rm{W}}}_{{\rm{i}}}-{{\rm{W}}}_{{\rm{r}}}){/{\rm{W}}}_{{\rm{i}}}$$

2 $${{\rm{W}}}_{{\rm{i}}}=({{\rm{C}}}_{{\rm{i}}}-{{\rm{C}}}_{{\rm{f}}})\times {{\rm{Q}}}_{{\rm{i}}}$$

3 $${{\rm{W}}}_{{\rm{r}}}={{\rm{C}}}_{{\rm{f}}}\times {{\rm{Q}}}_{{\rm{i}}}+{{\rm{C}}}_{{\rm{s}}}\times {{\rm{W}}}_{{\rm{s}}}$$

4 $${{\rm{R}}}_{{\rm{n}}}={{\rm{R}}}_{{\rm{t}}}-{{\rm{R}}}_{{\rm{c}}}$$

5 $$RGR=\frac{\mathrm{ln}\,{W}_{2}-\,\mathrm{ln}\,{W}_{1}}{{t}_{2}-{t}_{1}},$$

where, W_r_ (g) represents the average residual quantity (g) of nutrients in each pond; and W_i_ (g) is the initial total quantity (g) of the nutrient in each pond. C_i_ and C_f_ represent the initial and final concentrations (mg L^−1^) of TN and TP in the water column, respectively. Qi represents the volume (L) of the water in the experimental pond. Cs represents the concentration (mg g^−1^) of TN and TP in the sediment and Ws represents the mass (g) of the sediment in each pond. R_n_ (%) is the net removal rate of each treatment; R_t_ (%) is the total ultimate average removal rate in each treatment; R_c_ (%) represents the average removal rate of the control (group C); W_1_ and W_2_ represent the initial and final dry weights of the plants, respectively; and t_1_ and t_2_ represent the initial and final experimental time (days), respectively.

The mass balance of nitrogen and phosphorus in EFBs was calculated using the following equations:

6 $${{\rm{R}}}_{{\rm{total}}}={{\rm{R}}}_{{\rm{p}}}+{{\rm{R}}}_{{\rm{s}}}+{{\rm{R}}}_{{\rm{m}}}$$

7 $${{\rm{R}}}_{{\rm{p}}}={{\rm{C}}}_{{\rm{pf}}}\times {{\rm{W}}}_{{\rm{f}}}-{{\rm{C}}}_{{\rm{pi}}}\times {{\rm{W}}}_{{\rm{p}}}$$

8 $${{\rm{R}}}_{{\rm{s}}}=({{\rm{C}}}_{{\rm{sf}}}-{{\rm{C}}}_{{\rm{si}}})\times {{\rm{W}}}_{{\rm{ss}}}$$

where R_total_ represents the total removal quantity (g) of TN and TP during the experimental period, R_p_, R_s_ and R_m_ represents the total removal quantity (g) of N and P by plant uptake, substrate adsorption, and microbial degradation, respectively. C_pf_ and W_f_ represent the final concentration (mg g^−1^) of TN and TP within the plant and plant biomass (g DW), respectively. C_pi_ and W_p_ were the initial concentrations (mg g^−1^) of the N and P of the plant and plant biomass (g DW), respectively. Rs represents the total removal quantity of N and P by substrate adsorption in each pond; C_sf_ and C_si_ represent the final and initial concentration (g kg^−1^) of N and P of the substrate, respectively; and W_ss_ represents the total mass of the substrate in each pond (kg/pond).

Significant differences between treatments were determined by an analysis of variance (ANOVA) followed by post-hoc testing using Tukey’s HSD test. A P-value of less than 0.05 could be interpreted to declare that the differences were statistically significant. Statistical analyses were performed with SPSS 17.0 (SPSS Inc., Chicago, IL, USA).
